# Risk factors associated with loneliness among mexican-origin adults in southern Arizona

**DOI:** 10.1186/s12889-024-19199-x

**Published:** 2024-06-25

**Authors:** Mario Morales, Ada M. Wilkinson-Lee, Maia Ingram, Thomas Nuño, Jill E. Guernsey De Zapien, Ramses Sepulveda, Scott Carvajal

**Affiliations:** 1 Arizona Prevention Research Center, Health Promotion Sciences Department, Mel & Enid Zuckerman College of Public Health, The University of Arizona, Tucson, AZ 85724 USA; 2 Department of Mexican American Studies, The University of Arizona, Tucson, AZ 85721 USA

**Keywords:** Loneliness, Mexican-origin adults, Social determinants of health, US-Mexico border

## Abstract

This study examines factors associated with symptoms of loneliness among a sample (*n* = 213) of mostly Mexican-origin adults at risk of chronic diseases in Southern Arizona’s Pima, Yuma, and Santa Cruz counties. It uses baseline data from a community-based participatory research partnership and multinominal logistic regression models. Controlling for chronic diseases and sociodemographic characteristics, perceived social support and hope exhibit negative main effects on loneliness when comparing individuals who experienced loneliness for 5–7 days in the preceding week with those who did not encounter such feelings during the same period (adjusted odds ratio, AOR = 0.49 and 0.47; 95% confidence interval, CI = 0.34–0.73 and 0.29–0.75, respectively). However, when considered together, perceived social support and hope display a positive and statistically significant combined effect on loneliness (AOR = 1.03; 95% CI = 1.01–1.06). Holding all covariates constant, individuals reporting loneliness for 5–7 days exhibit a relative risk ratio of 1.24 (95% CI = 1.06–1.46) for a one-unit increase in physical problem severity compared to those who do not experience loneliness. Moreover, being 65 years old or older (AOR = 0.16, 95% CI = 0.03–0.84), and having been born in Mexico and lived in the US for less than 30 years (AOR = 0.12, 95% CI = 0.02–0.74) are associated with negative main effects on loneliness when comparing individuals who experienced loneliness 1–2, and 5–7 days in the preceding week with those who did not feel loneliness during the same timeframe, respectively. Recognizing the crucial role of loneliness in shaping health outcomes for Mexican-origin adults, our findings underscore the significance of fostering supportive environments that not only enhance well-being but also cultivate robust community bonds within the US-Mexico border region.

**Supplementary Information:**

The online version contains supplementary material available at 10.1186/s12889-024-19199-x.

## Background

Loneliness is a major risk factor for physical and mental health issues, as well as premature death, and has been documented worldwide [1–4]. Loneliness is a subjective feeling of being isolated, while social isolation refers to the objective state of having few social relationships, social roles, group memberships, or infrequent social contact with others [2, 3, 5]. This research investigates factors linked to self-reported loneliness symptoms within a population of mostly Mexican-origin adults vulnerable to chronic ailments residing in Pima, Yuma, and Santa Cruz counties in southern Arizona. The study posits that heightened loneliness correlates with diminished social support, hope, and heightened physical discomfort. Furthermore, the study anticipates an association between increased self-reported loneliness and self-reported instances of diabetes and depression. Recognizing the prevalence of loneliness among Mexican-origin adults across these three southern Arizona counties is pivotal for devising strategies and interventions to tackle this notable public health issue.

Globally, the prevalence of loneliness among adults varies from 5 to 43% [1]. Within the United States (US), three surveys conducted in 2018 yielded loneliness prevalence estimates ranging from 22 to 54% [6–10]. In the context of the COVID-19 pandemic, a CIGNA survey disclosed that 61% of the US populace acknowledged experiencing loneliness, with the Latine community reporting the highest level of loneliness at 47.7% [10]. A study involving a random sample of 755 elderly individuals from southern New Mexico, comprising 72% whites, 23% Latine (primarily of Mexican origin), and 5% from other ethnic backgrounds, revealed that Latine individuals exhibited greater perceived social isolation compared to whites. Notably, there were no substantial disparities in levels of loneliness between these two groups [11]. This study seeks to fill the gap in research investigating the connection between loneliness and health outcomes among a sample of mostly Mexican-origin adults in the US-Mexico border region. It defines Latine as an inclusive term covering a broader cultural and linguistic identity [12], and distinguishes Mexican-origin as a descriptor of origin within a specific geographical and temporal context.

Loneliness is associated with unfavorable cardiovascular health indicators, including high blood pressure and diabetes [13], elevated cholesterol levels [14], and coronary heart disease [15]. Furthermore, loneliness correlates with depression [16], anxiety [17], stress [18], sleep disturbance [19], physical inactivity [20], obesity [21], suicidal ideation [22], substance use [23], cognitive impairment, and dementia [24, 25]. A meta-analysis review (*n* = 70) revealed that reported loneliness was associated with a 26% increased likelihood of death [4]. Another meta-analysis (*n* = 31) found that the impact of loneliness on all-cause mortality was slightly higher for males than females [26].

Social support, encompassing the presence of individuals available to offer aid during challenging situations [27], functions as a safeguard against the onset of loneliness symptoms [28, 29]. Complementary to this, hope, denoting optimistic prospects for the future [30], likewise acts as a shield against loneliness symptoms [31]. Previous research has identified a positive correlation between social support and hope [32]. Individuals can embrace hope as an adaptive mechanism, utilizing it to generate a positive impact after accessing essential resources through the lens of perceived social support [33]. In challenging situations, hope functions as a positive coping mechanism or adaptive factor, while social support furnished the necessary coping resources [33]. However, the current understanding does not explore the role of hope as a moderating factor in the relationship between perceived social support and loneliness within the context of the US-Mexico border region. For instance, it may be plausible that Mexican-origin adults who maintain optimism and possess strong social ties are more likely to report fewer loneliness symptoms than their counterparts. Thus, this study hypothesizes that hope positively moderates the effect of social support on loneliness.

Physical illness and disability are positively associated with loneliness [34, 35]. This relationship can be understood through the lens of pain, which can hinder both physical activity and social interactions, consequently contributing to feelings of isolation.^40^ Furthermore, the distress stemming from loneliness can potentially exacerbate pain, creating a reciprocal relationship.^40^ Illustratively, within a sample of 741 community-dwelling adults situated in Phoenix, Arizona, pain intensity and frequency demonstrated a cross-sectional association with heightened loneliness, although this relationship was not echoed longitudinally [36]. Similarly, in the context of older adults, loneliness showed an association with the symptom grouping of pain, fatigue, and depression [37]. In light of these premises, the current study hypothesizes that a dearth of physical problem severity is positively related to loneliness.

Arizona notably boasts a higher proportion of Latine individuals, accounting for approximately 30% of the populace, 88% of whom trace their origins to Mexico [38, 39]. Given the challenging socioeconomic circumstances, Mexican-origin adults in Arizona encounter substantial impediments to healthcare access, further compounded for undocumented immigrants [40, 41]. This underscores the pertinence of investigating the pressing public health concerns of loneliness and social isolation [9, 42]. The imperative for conducting this study rests in the urgent need to comprehend and address the nuanced interplay of social, cultural, and health determinants within these distinctive contexts. By unraveling these intricate dynamics, we can inform targeted interventions, foster well-being, enhance support systems, and ultimately enrich the quality of life for this underserved demographic.

## Methods

### Study and intervention

This study was initiated as part of the Linking Individuals Needs to Community and Clinical Services (LINKS) project, employing a prospective matched observational study design integrated with electronic health records, as previously detailed [43]. In the LINKS project, clinic-based community health workers (CHWs) in three federally qualified health centers (FQHCs) referred eligible patients to community-based CHWs in county health department using a project REDcap database. The community-based CHWs enrolled the participant in the study and conducted baseline surveys in either English or Spanish. Beyond survey administration, the community-based CHWs identified participants’ needs related to the social determinants of health, facilitated referrals to relevant programs and services, and provided guidance on emotional well-being techniques [43]. Notably, the FQHCs are pivotal providers of primary care to uninsured and underinsured populations, serving as cornerstones within the study counties.

### Sample and setting

The study sample encompassed a sample (*n* = 213) of mostly Mexican-origin adults who fulfilled baseline assessment requirements. Inclusion criteria necessitated a minimum age of 21, proficiency in either English or Spanish, and being at risk of or afflicted by a chronic disease, as indicated by individual electronic health records containing weight, height, body mass index, glycosylated hemoglobin, blood pressure, and blood lipid profile data [43]. Participants engaged with CHWs at one of three federally qualified health centers—located in Pima, Yuma, or Santa Cruz County—to complete baseline surveys in either English or Spanish. This data collection transpired between July 2017 and September 2018, with CHWs securing written consent from all participants enrolled in the LINKS initiative. The research was conducted in accordance with the approval granted by the University of Arizona Institutional Review Board (1612044741R001).

### Questionnaire

Conceived as a community-based participatory research endeavor, the LINKS project emerged through partnership between an academic institution and community collaborators encompassing health centers, county health departments, and other community health advocates. This dynamic partnership extended across the entire spectrum of the study, notably including the development of the Emotional Well-being Questionnaire. This questionnaire included adaptations (for language and local responsiveness) of the Social Support Inventory—Enhancing Recovery in Coronary Heart Disease (SSI-ENRICHED) [44], the State Hope Scale (SHS) [45], the Short Form 8 Health Survey (SF-8) [46], and sociodemographics [43]. The dependent variable (i.e., loneliness) was measured using the following statement/question: I felt lonely during the past week; response categories scored 1 = less than 1 day, 2 = 1–2 days, 3 = 3–4 days, and 4 = 5–7 days. The supplemental material also includes the results of four logit models running loneliness as a binary variable: 0 = less than 1 day, and 1 = 1–7 days.

Comprising six items, the Emotional Well-being Questionnaire gauged perceptions of social support over the preceding four weeks. Participants responded to prompts such as “Is there someone available to you whom you can count on to listen to you when you need to talk?”, “Is there someone available to give you good advice about a problem?”, “Is there someone available to you who shows you love and affection?”, “Is there someone available to help you with daily chores?”, “Can you count on anyone to provide you with emotional support?”, and “Do you have as much contact as you would like with someone you feel close to, someone in whom you can trust and confide?” Each question’s response options were grouped as follows: scale was reversed, where 1 = none of the time, 2 = a little of the time, 3 = some of the time, 4 = most of the time, and 5 = all the time. The cumulative scores, ranging from 1 to 24, furnished insight into the level of social support, with higher scores denoting greater support. Notably, the measure exhibited robust reliability within this sample, with a Cronbach’s α of 0.856 (95% CI: 0.817, 0.884).

Hope was assessed through six items that explored participants’ present agency and pathways toward achieving their objectives. That is, “If I should find myself in a jam, I could think of many ways to get out of it”, “At the present time, I am energetically pursuing my goals”, “There are lots of ways around any problem that I am facing now”, “Right now I see myself as being pretty successful”, “I can think of many ways to reach my current goals”, and “At this time, I am meeting the goals that I have set for myself”. Each item’s response options were grouped as follows: scale was reversed, where 1 = none of the time, 2 = a little of the time, 3 = some of the time, 4 = most of the time, and 5 = all the time. Scores were aggregated, resulting in a range of 1 to 20, wherein higher scores were indicative of heightened hopefulness. Encouragingly, the reliability coefficient for this sample was robust (Cronbach’s α = 0.879; 95% CI: 0.844, 0.905).

The physical problem severity index was assessed using four items that considered the previous four weeks. Participants were asked about the extent to which physical health issues constrained their physical activities, the challenges they faced in performing daily tasks at home and outside due to their physical health, the level of bodily pain experienced, and how their physical or emotional health impacted their usual social interactions with family and friends. For items pertaining to limitations on physical activities, response options were 1 = not at all, 2 = very little, 3 = somewhat, 4 = quite a lot, and 5 = could not do daily work. On the other hand, for items addressing bodily pain, answer choices ranged from 1 = none to 6 = very severe. Cumulative scores ranged from 1 to 18, with higher scores denoting greater severity of physical problems. The measure exhibited robust reliability within this sample, boasting a Cronbach’s α of 0.848 (95% CI: 0.811, 0.878).

Self-reported diabetes and depression were gauged through a single question: “Do you have any of the following health problems—diabetes and depression?” Participants responded with 0 for no and 1 for yes. Control variables, representing sociodemographic factors, were measured as follows: Age was captured as a categorical variable: 0 < 45, 1 = 45–64, 2 = 65+. Gender was denoted as a binary variable, with 0 signifying male and 1 signifying female. Education was categorized into 0 for < 12 years and 1 for ≥ 12 years of education. Time lived in the US was categorized into three groups: 0 for being born in the US, 1 for being born in Mexico and residing in the US for ≤ 30 years, and 2 for being born in Mexico and residing in the US for > 30 years. The delineation aimed to ensure a balanced sample distribution. County was depicted as a nominal variable with values of 0 for Pima, 1 for Yuma, and 2 for Santa Cruz, reflecting the respective study locales.

The Arizona Prevention Research Center (AzPRC) closely collaborated with the enduring 25-year Community Action Board, instrumental in selecting study questionnaires and shaping research questions [47]. Adaptation, driven by community needs, aimed to capture their unique viewpoints. In enhancing measurement tools, research partners adjusted instruments to suit the intervention context [48]. AzPRC’s translation process employed a functional adequacy approach, consensually defining each question’s functional meaning, deviating from the traditional back-translation [49]. This method ensured high-quality translations, vital in cross-cultural research. For example, eight items from the SF-8 quality-of-life instrument were adapted and translated for LINKS participants to reflect local culture and language nuances [49]. In contrast to the standard SF-8 in English, the adapted instrument lacked normative values, representing quality of life by summing responses from the eight items [49].

### Data analysis

Within the study, missing data, which accounted for less than 5% across all variables, were addressed through predictive mean matching imputation. This method involved replacing missing values with observed donor values through the utilization of the Mice package in R 4.1 [50]. A total of five imputations were generated and subsequently merged with the original observed dataset. To compare Mexican-origin individuals with and without self-reported loneliness, encompassing perceived social support, hope, self-reported diseases, and demographic traits, descriptive statistics were employed. The Mann–Whitney U test was utilized for analysis, and statistical significance was established at α = 0.05. To investigate the determinants of self-reported loneliness while adjusting for self-reported diseases and sociodemographic attributes, multinomial and logit regression models were employed. Potential moderator variables were identified through scrutiny of interaction terms, with their inclusion in the final model contingent on statistical significance at a *p* value < 0.05. Model fit and comparison between nested models were assessed using the log likelihood test statistic. The entirety of the data analysis was executed using R software, with specific reliance on version R 4.1.

## Results

Table 1 presents a succinct overview of the baseline attributes within the analyzed sample. Predominantly, participants identified as female (85.9%), with the majority falling within the age range of 45 to 64 (49.8%). Slightly over half completed up to 12 years of education (57.3%). Only 16.9% were born in the US, while 34.7% had resided in the US for at least three decades. Within the total sample, 58.7% indicated not experiencing loneliness during the prior week. However, 11.7% reported feeling lonely for a minimum of 5 days in that same period. The average participant reported elevated levels of both social support (scoring 17.8 within a range of 1 to 24) and hope (scoring 14.5 within a range of 1 to 20). In contrast, participants signaled relatively lower levels of physical problem severity (averaging 6.56 within a range of 1 to 18). Notably, 39.4% and 26.8% of respondents reported having diabetes and depression, respectively.

**Table 1:** Baseline characteristics of the links sample (multinomial dependent variable) *

	0 (*N* = 128)	1–2 (*N* = 38)	3–4 (*N* = 22)	5–7 (*N* = 25)	Total (*N* = 213)	NA (4)	Mann-Whitney U test
**Physical Pain**							
Mean (SD)	5.58 (4.17)	6.74 (3.90)	8.77 (4.70)	9.40 (4.89)	6.56 (4.48)	5	< 0.001
Median [Min, Max]	4.00 [1.00, 15.0]	7.00 [1.00, 16.0]	8.00 [1.00, 17.0]	9.00 [1.00, 18.0]	6.00 [1.00, 18.0]		
**Social Support**							
Mean (SD)	20.0 (4.11)	15.6 (5.81)	15.9 (5.73)	11.9 (6.50)	17.8 (5.68)	3	< 0.001
Median [Min, Max]	21.0 [7.00, 24.0]	17.0 [1.00, 24.0]	17.5 [5.00, 24.0]	11.0 [1.00, 24.0]	19.0 [1.00, 24.0]		
**Hope**							
Mean (SD)	16.1 (3.40)	14.3 (4.27)	12.1 (5.21)	8.80 (5.40)	14.5 (4.69)	10	< 0.001
Median [Min, Max]	16.5 [2.00, 20.0]	14.5 [4.00, 20.0]	13.0 [2.00, 20.0]	7.00 [1.00, 20.0]	15.0 [1.00, 20.0]		
**Age (years)**							
18–44	22 (17.2%)	9 (23.7%)	2 (9.1%)	5 (20.0%)	38 (17.8%)	1	0.078
45–64	55 (43.0%)	23 (60.5%)	14 (63.6%)	14 (56.0%)	106 (49.8%)		
65+	51 (39.8%)	6 (15.8%)	6 (27.3%)	6 (24.0%)	69 (32.4%)		
**Sex**							
Male	17 (13.3%)	6 (15.8%)	5 (22.7%)	2 (8.0%)	30 (14.1%)	0	0.518
Female	111 (86.7%)	32 (84.2%)	17 (77.3%)	23 (92.0%)	183 (85.9%)		
**Education (years)**							
<12	67 (52.3%)	22 (57.9%)	19 (86.4%)	14 (56.0%)	122 (57.3%)	8	0.031
>=12	61 (47.7%)	16 (42.1%)	3 (13.6%)	11 (44.0%)	91 (42.7%)		
**Place of Birth/Years in US**							
US Birth	21 (16.4%)	6 (15.8%)	3 (13.6%)	7 (28.0%)	37 (17.4%)	7	0.542
MX Birth and US < = 30	65 (50.8%)	20 (52.6%)	8 (36.4%)	9 (36.0%)	102 (47.9%)		
MX Birth and US > 30	42 (32.8%)	12 (31.6%)	11 (50.0%)	9 (36.0%)	74 (34.7%)		
**County**							
Pima	50 (39.1%)	29 (76.3%)	8 (36.4%)	6 (24.0%)	93 (43.7%)	0	< 0.001
Yuma	46 (35.9%)	5 (13.2%)	7 (31.8%)	12 (48.0%)	70 (32.9%)		
Santa Cruz	32 (25.0%)	4 (10.5%)	7 (31.8%)	7 (28.0%)	50 (23.5%)		
**Diabetes**							
No	80 (62.5%)	24 (63.2%)	7 (31.8%)	18 (72.0%)	129 (60.6%)	0	0.026
Yes	48 (37.5%)	14 (36.8%)	15 (68.2%)	7 (28.0%)	84 (39.4%)		
**Depression**							
No	100 (78.1%)	30 (78.9%)	16 (72.7%)	10 (40.0%)	156 (73.2%)	0	0.001
Yes	28 (21.9%)	8 (21.1%)	6 (27.3%)	15 (60.0%)	57 (26.8%)		

* NA refers to missing data in each variable of the original dataset. However, all descriptive statistics correspond to the imputed data

Table 2 displays four multinomial logistic regression models, utilizing imputed data to explore the correlates of loneliness symptoms while adjusting for various health scales, chronic diseases and sociodemographic factors. The “Scales of Health and Chronic Diseases” model encompasses the scales of physical pain, social support and hope, along with chronic diseases such as diabetes and depression. The “Sociodemographics” model controls for age, sex, education, place of birth/years in the US, and county. The “Full Model” incorporates all previous scales, diseases, and sociodemographics. Finally, the “Full Model with Interaction Effect” introduces the combined effect of social support and hope into the previous model. The last model aims to uncover the nuanced associations between social support and loneliness, considering different levels of hope. After controlling for chronic diseases and sociodemographic characteristics, the final model (displayed in the last column) reveals significant findings. Perceived social support and hope demonstrate negative main effects on loneliness when comparing individuals who experienced loneliness for 5–7 days in the preceding week with those who did not feel lonely during the same period (adjusted odds ratio, AOR = 0.49 and 0.47; 95% confidence interval, CI = 0.34–0.73 and 0.29–0.75, respectively). However, the combination of perceived social support and hope displays a positive and statistically significant effect on loneliness (AOR = 1.03; 95% CI = 1.01–1.06). When holding all covariates constant, individuals reporting loneliness for 5–7 days exhibit a relative risk ratio of 1.24 (95% CI = 1.06–1.46) for a one-unit increase in physical problem severity compared to those who do not experience loneliness. Additionally, being 65 years old or older (AOR = 0.16, 95% CI = 0.03–0.84), and being born in Mexico and having lived in the US for less than 30 years (AOR = 0.12, 95% CI = 0.02–0.74) show negative main effects on loneliness when comparing individuals who experienced loneliness 1–2, and 5–7 days in the preceding week with those who did not feel loneliness during the same period, respectively. Notably, variables including diabetes, depression, sex, and education do not exhibit significant main effects or interaction effects on loneliness symptoms at a *p* value < 0.05.

**Table 2:** Multinomial logistic regression models of the determinants of self-reported loneliness over the past week among mexican-origin adults in Pima, Yuma, and Santa Cruz, Arizona

	Days	Scales & Diseases / Est.	Confidence Interval	Sociodemo. / Est.	Confidence Interval	Full Model / Est.	Confidence Interval	Full Model with Interaction Effect / Est.	Confidence Interval
Social Support	1–2	0.85 ***	[0.78, 0.92]			0.81 ***	[0.74, 0.89]	0.70 *	[0.50, 0.98]
	3–4	0.88 *	[0.80, 0.97]			0.86 **	[0.78, 0.96]	0.80	[0.58, 1.10]
	5–7	0.80 ***	[0.72, 0.89]			0.75 ***	[0.66, 0.85]	0.49 ***	[0.34, 0.73]
Hope	1–2	0.93	[0.84, 1.03]			0.90 +	[0.80, 1.02]	0.75	[0.51, 1.11]
	3–4	0.83 **	[0.74, 0.94]			0.84 *	[0.73, 0.96]	0.75	[0.50, 1.13]
	5–7	0.79 ***	[0.70, 0.89]			0.79 **	[0.68, 0.91]	0.47 **	[0.29, 0.75]
Physical Pain	1–2	1.05	[0.96, 1.16]			1.10	[0.97, 1.24]	1.10	[0.98, 1.24]
	3–4	1.13 +	[1.00, 1.27]			1.13	[0.97, 1.31]	1.13	[0.97, 1.31]
	5–7	1.16 *	[1.02, 1.32]			1.19 *	[1.03, 1.39]	1.24 **	[1.06, 1.46]
Diabetes (Yes)	1–2	0.89	[0.39, 2.03]			1.62	[0.58, 4.49]	1.68	[0.60, 4.71]
	3–4	3.28 *	[1.13, 9.49]			3.20 +	[0.92, 11.16]	3.59 +	[0.97, 13.21]
	5–7	0.36	[0.10, 1.34]			0.38	[0.09, 1.64]	0.29	[0.06, 1.44]
Depression (Yes)	1–2	0.60	[0.21, 1.68]			0.77	[0.24, 2.54]	0.73	[0.22, 2.46]
	3–4	0.43	[0.12, 1.53]			0.47	[0.11, 1.96]	0.46	[0.11, 2.02]
	5–7	1.55	[0.45, 5.36]			1.73	[0.46, 6.58]	1.44	[0.35, 6.02]
Age (45–64 years)	1–2			0.93	[0.33, 2.60]	0.97	[0.31, 3.07]	0.91	[0.29, 2.91]
	3–4			1.98	[0.34, 11.52]	2.09	[0.30, 14.67]	2.12	[0.29, 15.36]
	5–7			1.16	[0.31, 4.30]	1.57	[0.25, 9.86]	1.07	[0.17, 6.70]
Age (65 + years)	1–2			0.20 *	[0.05, 0.85]	0.17 *	[0.03, 0.91]	0.16 *	[0.03, 0.84]
	3–4			0.32	[0.04, 2.54]	0.26	[0.02, 2.77]	0.24	[0.02, 2.72]
	5–7			0.36	[0.08, 1.72]	0.78	[0.09, 6.70]	0.57	[0.07, 4.70]
Sex (Female)	1–2			0.80	[0.25, 2.53]	0.90	[0.23, 3.47]	0.88	[0.22, 3.48]
	3–4			0.45	[0.13, 1.61]	0.37	[0.08, 1.68]	0.36	[0.07, 1.72]
	5–7			1.74	[0.35, 8.61]	1.68	[0.21, 13.21]	1.29	[0.16, 10.41]
Education ( > = 12 years)	1–2			0.56	[0.24, 1.28]	0.99	[0.38, 2.60]	0.91	[0.34, 2.42]
	3–4			0.13 **	[0.03, 0.50]	0.23 +	[0.05, 1.07]	0.22 +	[0.04, 1.05]
	5–7			0.67	[0.26, 1.71]	1.85	[0.47, 7.39]	1.66	[0.39, 7.02]
MX Birth and US < = 30 years	1–2			0.56	[0.17, 1.90]	0.56	[0.14, 2.24]	0.55	[0.13, 2.24]
	3–4			0.49	[0.10, 2.47]	0.83	[0.13, 5.42]	0.95	[0.13, 6.77]
	5–7			0.38	[0.11, 1.31]	0.16 *	[0.03, 0.99]	0.12 *	[0.02, 0.74]
MX Birth and US 30 + years	1–2			1.61	[0.42, 6.15]	1.56	[0.35, 6.90]	1.72	[0.38, 7.86]
	3–4			1.46	[0.27, 7.94]	1.98	[0.30, 13.12]	2.21	[0.32, 15.31]
	5–7			0.78	[0.20, 2.99]	0.31	[0.05, 1.96]	0.30	[0.05, 1.92]
County (Yuma)	1–2			0.16 **	[0.05, 0.47]	0.06 ***	[0.02, 0.23]	0.06 ***	[0.01, 0.22]
	3–4			0.59	[0.17, 2.05]	0.19 *	[0.04, 0.86]	0.18 *	[0.04, 0.85]
	5–7			1.83	[0.59, 5.62]	0.29	[0.06, 1.35]	0.36	[0.08, 1.69]
County (Santa Cruz)	1–2			0.25 *	[0.07, 0.89]	0.18 *	[0.04, 0.80]	0.18 *	[0.04, 0.79]
	3–4			1.72	[0.46, 6.38]	1.07	[0.22, 5.24]	1.09	[0.21, 5.52]
	5–7			1.97	[0.53, 7.25]	0.53	[0.08, 3.55]	0.46	[0.06, 3.28]
Social Support: Hope	1–2							1.01	[0.99, 1.03]
	3–4							1.00	[0.98, 1.03]
	5–7							1.03 *	[1.01, 1.06]
N		213		213		213		213	
R2		0.23		0.12		0.34		0.35	

+ *p* < 0.1, * *p* < 0.05, ** *p* < 0.01, *** *p* < 0.001

## Discussion

This study harnessed cross-sectional data centered on Mexican-origin adults at risk of chronic disease within three Arizona border counties to scrutinize the correlates of loneliness symptoms. These findings contribute to an enhanced understanding of the intricate relationships shaping loneliness symptoms within this study’s context. Approximately 40% of respondents reported experiencing loneliness for at least one day in the preceding week. This prevalence aligns with both international and national reports on loneliness symptoms [1, 6–10, 51]. The statistical examination yielded intriguing insights: the interplay between social support and loneliness symptoms could be influenced by participants’ hope levels. Specifically, the combined effect of heightened hope and social support was linked to elevated reports of loneliness symptoms. Additionally, a noteworthy positive association emerged between physical problem severity index and loneliness symptoms. Interestingly, variables such as diabetes, depression, sex, and education exhibited no statistically significant associations with self-reported loneliness among participants. Nevertheless, these aspects remain vital considerations for comprehending the study’s focal outcome. This study extends the existing body of research by incorporating confirmatory factor analysis for primary independent variables, incorporating imputations for addressing missing data, and employing multinomial logistic regression models to dissect explanatory factors. The findings underscore the persistent necessity for holistic prevention and treatment healthcare interventions tailored to tackle the distinct challenges posed by loneliness. The insights garnered here propel us toward more effective strategies for addressing the multifaceted impacts of loneliness within vulnerable populations.

Contrary to existing findings suggesting that heightened perceived social support and hope correlate with reduced loneliness [32, 33], this study presents a divergence. It is important to recognize that perceived social support may not automatically translate into immediate access to coping mechanisms, thus potentially necessitating the adoption of hopeful thinking as a coping strategy. The interplay between the nature of support (e.g., directive vs. non-directive or synthetic vs. instinctive) and support provider matters (e.g., clinicians and CHW vs. family and friends) [48]. A previous report identified four cluster narratives of support within this population: those with high emotional but minimal tangible/informational assistance; those with high emotional support but low appraisal/informational assistance; those with high informational assistance but low emotional/appraisal support; and those with a balanced mix of emotional, appraisal and informational support [48]. The noteworthy negative and significant interaction effect of social support and hope on loneliness warrants a cautious interpretation. Social support typically nurtures a sense of optimism about life [29], which one would anticipate having a beneficial impact on loneliness symptoms. Paradoxically, this study identifies a small but statistically significant association in the opposite direction: greater social support and hope corresponded with heightened loneliness symptoms. It is possible that a more nuanced approached to quantifying social support, breaking it down into different types, may shed light on this paradoxical results. Likewise, it is plausible that individuals who have family history of life-threatening diseases and long disease duration had low levels of hope and social support [52]. This underscores the complexity of these relationships and highlights the necessity of further research to unravel these intricate dynamics and their potential implications for intervention strategies.

Social support, encompassing cultural values such as familismo and respeto, can prove instrumental in alleviating loneliness symptoms [48]. Given the reinforcement of collectivism and interpersonal bonds in Mexican culture [53], Mexican-origin adults might rely significantly on social relationships to navigate daily stressors [54, 55]. Furthermore, familial and friend networks could serve as pathways to accessing formal social services [56]. However, given the multidimensional nature of social support, forthcoming research could profitably investigate various forms and sources of support. This could encompass tangible, instrumental, and emotional support, alongside assistance from partners, family, friends, colleagues, neighbors, and pets [11]. Likewise, hope’s potential to activate mechanisms that moderate psychological distress is noteworthy. Future inquiries should discern between two closely linked patterns of hopeful thinking: agentic and pathway thinking. The former delves into the driving force behind defining and achieving goals, while the latter involves formulating strategies to navigate obstacles and achieve success [31–33].

These findings hold significant policy and practice implications. Firstly, healthcare interventions should integrate hope-centric approaches, especially when social support diminishes due to the evolving dynamics of caring for older adults or the growing burden of their illnesses on families [52]. This integration may involve fostering optimistic thinking and resilience. Secondly, in contexts like the US-Mexico border region in Southern Arizona, characterized by strong familial bonds, community-based efforts should explore hope as a communal asset. Strategies such as collaborative projects, support networks, and community engagement can build resilience and foster hope. Lastly, the positive association between satisfaction with health information from professionals and hope suggests the potential efficacy of educational interventions [57]. Viewing hope as a dynamic resource guides the development of interventions enhancing individuals’ ability to navigate challenges and cultivate connectedness within social networks. Recognizing hope as a valuable asset allows interventions to foster optimism, resilience, and ultimately alleviate loneliness in diverse populations.

Prior research has illuminated correlations between baseline loneliness and subsequent moderate to intense pain, as well as the reciprocal link where baseline pain is connected to later loneliness [35]. Nonetheless, a more comprehensive exploration is warranted to discern the intricate interplay between pain intensity and loneliness, particularly within specific populations marked by frequent pain [36]. Future inquiries could also delve into potential moderating factors such as negative mood, depression, fatigue, or anger to uncover their impact on the longitudinal relationship between pain and loneliness [36]. This paradigm extends to the consideration of symptom clusters, including pain, depression, and fatigue, which are recognized within distinct populations, such as those grappling with multiple sclerosis, fibromyalgia, and cancer [58]. Given this context, it becomes imperative for forthcoming research to not only probe the linkage between loneliness and these symptoms individually but also investigate their collective manifestation. As such, a comprehensive exploration of the interconnected distress these factors pose is crucial to advancing our understanding of loneliness within these contexts.

Although depression and loneliness both reside within the realm of cognitive-psychosocial states [37], their interrelationship did not yield statistical significance. Similarly, no significant association emerged between diabetes and loneliness. An investigation conducted in Tucson during 2013–2014, involving interviews with 32 first-generation Mexican immigrants, underscored that a considerable 72.5% of participants felt lonely due to their legal status and a lack of community support [59]. Similarly, among 39 immigrants receiving legal services in western Texas and New Mexico in 2015, 38.5% reported grappling with feelings of loneliness and isolation within the US [60]. This study detected a negative and statistically significant association between loneliness, and being born in Mexico and having lived in the US for less than 30 years. This may potentially be attributed to the fact that a substantial proportion of participants born outside the US had accumulated significant years of residency within the country. The noteworthy negative and statistically significant association observed between experiencing loneliness for 1–2 days and being 65 years old or older underscores a crucial point: both younger and older adults encounter loneliness. Despite this, societal attention has primarily concentrated on loneliness among older adults [14]. Therefore, it becomes imperative to redirect our focus towards addressing loneliness experienced during middle ages within this specific demographic residing in the US-Mexico border region.

Research has identified practices that wield positive effects in alleviating loneliness. These encompass well-informed intergenerational program designs, which can incorporate elements such as technological integration, environmental enhancements, comprehensive training for both facilitators and participants, and fostering high-quality interactions among participants through mechanisms that foster friendship [61]. The potential interventions within an intergenerational framework can encompass diverse strategies, including one-to-one engagements, activities conducted in group settings, technology-driven initiatives, and hybrid approaches that blend these methods [5]. To engender effective intergenerational programs, practitioners should emphasize crafting an environment that is both secure and supportive. This should be coupled with the cultivation of consistent and positive adult-adolescent relationships, developmentally suitable programming, acknowledgment and celebration of the cultural and social influences on adolescent growth, and the provision of opportunities for both older adults and youth to exercise autonomy, empowerment, self-direction, responsibility, and self-awareness [62]. By conscientiously weaving these elements together, practitioners can pave the way for the successful execution of intergenerational programs that contribute to combatting loneliness.

The National Academies of Science, Engineering, and Medicine have recommended the integration of education and training concerning social isolation and loneliness within the healthcare workforce [3]. As integral members of this workforce, CHWs are frontline public health professionals who possess profound insights into and trust within the communities they serve [38, 63, 64]. Their distinctive position empowers them to serve as a pivotal bridge between health and social services and the broader community [38, 63, 64]. Moreover, they have demonstrated the potential to cultivate social connectedness and enhance health outcomes [38, 63, 64]. Given the active involvement of the community health workforce in addressing mental health matters and their achievements in fostering patient rapport, it is imperative to assess their potential contribution in identifying loneliness and social isolation among both older adults and youth within the US-Mexico border region.

The cross-sectional data derived from the LINKS study present a blend of strengths and limitations. Among the prominent strengths lies its specific focus on a high-priority population residing within an underserved locale. Notably, the study adopts a model involving CHWs, who play a pivotal role in establishing links between participants and diverse health and social services. Furthermore, the research is enriched by its profound and continuous engagement with community organizations, ensuring valuable insights that inform all facets of the study. Additionally, the missing data represents less than 5% of the total sample. However, it is important to acknowledge several limitations. Especially, the target population and sample are somewhat homogenous. The findings primarily pertain to Mexican-origin adult females who attend community health clinics in Yuma, Pima, and Santa Cruz, Arizona. Hence, future research could consider diversifying the sample to include more males and potentially comparing a balanced sample of Mexican-origin and non-Mexican-origin individuals who access community health clinics versus those seeking healthcare elsewhere, or not at all, within the US–Mexico border region. Moreover, research indicates that delving into different facets of loneliness/social support or exploring distinct dimensions of loneliness/social support using specialized scales might furnish additional explanatory capabilities in forecasting loneliness symptoms among Mexican-origin adults [34, 48].

This study explored a particular arrangement of the loneliness, social support, and hope variables, yet alternative configurations remain plausible. For instance, a prior study identified that perceived social support was linked to heightened hope exclusively through a reduction in loneliness [32]. Further analyses are required to unveil the intricate mechanisms interconnecting these three variables, which could ideally involve longitudinal investigations. Moving forward, research should adopt longitudinal and experimental designs to illuminate the enduring predictive role of perceived social support and hope in relation to loneliness. Similarly, it is prudent for future studies to account for additional factors contributing to loneliness symptoms, such as medication usage, mental health history, and other life stressors. Within the context of the US–Mexico border region, it is equally vital to expand measurements to incorporate the structural determinants of health. For instance, it is essential to comprehend how social stressors, including perceived discrimination and repeated exposure to discriminatory situations, might directly or indirectly influence loneliness symptoms through various psychological and physiological responses. This holistic approach would significantly enhance the comprehensiveness of our understanding of loneliness in this specific context.

## Conclusion

The exploration of loneliness within the Mexican-origin population residing in the US-Mexico border region is of profound significance, bridging an enduring gap in the provision of mental health services for this historically excluded community. By embarking on this quest to rectify knowledge voids, this research endeavor carries substantial merit in advancing our comprehension of the intricate deleterious impact of loneliness on health outcomes, particularly within the complex context of rural and border communities. This effort paves the way for targeted intergenerational interventions and robust support frameworks that possess the potential to profoundly enhance the well-being of these underserved populations. In this endeavor, CHWs stand poised as instrumental agents capable of furnishing culturally attuned services within ethnic and language enclaves that grapple with constrained access to resources. Their pivotal role extends to the imperative task of disseminating information regarding available resources in a manner that is rooted in cultural sensitivities and contextual relevance. Given the indispensable role that social support and hope assume as vital resources for those navigating the challenges of loneliness, the pursuit of strategies geared toward bolstering these aspects gains even more prominence within the unique context of the US-Mexico border region.

### Electronic supplementary material

Below is the link to the electronic supplementary material.

Supplementary Material 1

## Data Availability

The datasets used and/or analyzed during the current study are available from the corresponding author on reasonable request.
