# Supplementary material for: Risk factors associated with loneliness among mexican-origin adults in southern Arizona
Source: BMC Public Health. 2024 Jun 25;24:1694. doi: 10.1186/s12889-024-19199-x (PMC11197176; doi:10.1186/s12889-024-19199-x)
Supplement: Supplementary file 1 — Supplementary Material 1 [file 12889_2024_19199_MOESM1_ESM.docx]

Supplementary Table 1. Baseline characteristics of the LINKS sample (Binary dependent variable)*

| **Loneliness** | 0 (N=128) | 1-7 (N=85) | Total (N=213) | P-value | NA  (4) |
| --- | --- | --- | --- | --- | --- |
| **Physical Pain** |  |  |  |  |  |
| Mean (SD) | 5.55 (4.15) | 8.00 (4.55) | 6.53 (4.47) | <0.001 | 5 |
| Median [Min, Max] | 4.00 [1.00, 15.0] | 8.00 [1.00, 18.0] | 6.00 [1.00, 18.0] |  |  |
| **Social Support** |  |  |  |  |  |
| Mean (SD) | 5.00 (4.11) | 10.4 (6.18) | 7.17 (5.69) | <0.001 | 3 |
| Median [Min, Max] | 4.00 [1.00, 18.0] | 9.00 [1.00, 24.0] | 6.00 [1.00, 24.0] |  |  |
| **Hope** |  |  |  |  |  |
| Mean (SD) | 5.61 (4.02) | 10.3 (6.48) | 7.48 (5.63) | <0.001 | 10 |
| Median [Min, Max] | 5.00 [1.00, 23.0] | 9.00 [1.00, 23.0] | 7.00 [1.00, 23.0] |  |  |
| **Age (years)** |  |  |  |  |  |
| 18-44 | 22 (17.2%) | 16 (18.8%) | 38 (17.8%) | 0.0355 | 1 |
| 45-64 | 55 (43.0%) | 51 (60.0%) | 106 (49.8%) |  |  |
| 65+ | 51 (39.8%) | 18 (21.2%) | 69 (32.4%) |  |  |
| **Sex** |  |  |  |  |  |
| Male | 17 (13.3%) | 13 (15.3%) | 30 (14.1%) | 0.125 | 0 |
| Female | 111 (86.7%) | 72 (84.7%) | 183 (85.9%) |  |  |
| **Education (years)** |  |  |  |  |  |
| <12 | 67 (52.3%) | 55 (64.7%) | 122 (57.3%) | 0.125 | 8 |
| >=12 | 61 (47.7%) | 30 (35.3%) | 91 (42.7%) |  |  |
| **Place of Birth/Years in US** |  |  |  |  |  |
| US Birth | 21 (16.4%) | 15 (17.6%) | 36 (16.9%) | 0.0313 | 7 |
| MX Birth and US <=30 | 65 (50.8%) | 38 (44.7%) | 103 (48.4%) |  |  |
| MX Birth and US >30 | 42 (32.8%) | 32 (37.6%) | 74 (34.7%) |  |  |
| **County** |  |  |  |  |  |
| Pima | 50 (39.1%) | 43 (50.6%) | 93 (43.7%) | 0.0313 | 0 |
| Yuma | 46 (35.9%) | 24 (28.2%) | 70 (32.9%) |  |  |
| Santa Cruz | 32 (25.0%) | 18 (21.2%) | 50 (23.5%) |  |  |
| **Diabetes** |  |  |  |  |  |
| No | 80 (62.5%) | 49 (57.6%) | 129 (60.6%) | 0.125 | 0 |
| Yes | 48 (37.5%) | 36 (42.4%) | 84 (39.4%) |  |  |
| **Depression** |  |  |  |  |  |
| No | 100 (78.1%) | 56 (65.9%) | 156 (73.2%) | 0.125 | 0 |
| Yes | 28 (21.9%) | 29 (34.1%) | 57 (26.8%) |  |  |

* Loneliness = Feeling lonely in the last week (0 = 0 days, and 1 = 1-7 days). The NA refers to missing data in each variable of the original dataset. However, all descriptive statistics correspond to the imputed data.

Supplementary Table 2. Binary logistic regression models of the determinants of self-reported loneliness over the past week among Mexican-origin adults in Pima, Yuma, and Santa Cruz, Arizona

|  | Scales & Diseases | Sociodemographics | Full Model | Full Model With Interaction |
| --- | --- | --- | --- | --- |
| Social Support | 0.17 *** |  | 0.20 *** | 0.15 * |
|  | [0.10, 0.24] |  | [0.12, 0.28] | [0.02, 0.28] |
| Hope | 0.12 *** |  | 0.14 *** | 0.10 |
|  | [0.05, 0.19] |  | [0.06, 0.23] | [-0.02, 0.23] |
| Physical Pain | 0.09 * |  | 0.12 * | 0.13 ** |
|  | [0.01, 0.17] |  | [0.03, 0.22] | [0.03, 0.23] |
| Diabetes (Yes) | 0.11 |  | 0.50 | 0.53 |
|  | [-0.57, 0.79] |  | [-0.33, 1.35] | [-0.31, 1.39] |
| Depression (Yes) | -0.36 |  | -0.11 | -0.18 |
|  | [-1.21, 0.45] |  | [-1.04, 0.80] | [-1.14, 0.74] |
| Age (45-64 years) |  | 0.10 | 0.08 | 0.03 |
|  |  | [-0.73, 0.95] | [-0.91, 1.09] | [-0.96, 1.04] |
| Age (65+ years) |  | -1.30 * | -1.44 * | -1.52 * |
|  |  | [-2.37, -0.28] | [-2.79, -0.16] | [-2.89, -0.23] |
| Sex (Female) |  | -0.16 | -0.40 | -0.38 |
|  |  | [-1.01, 0.71] | [-1.52, 0.74] | [-1.51, 0.76] |
| Education (>=12 years) |  | -0.81 * | -0.13 | -0.17 |
|  |  | [-1.45, -0.19] | [-0.94, 0.68] | [-0.99, 0.64] |
| MX Birth and US <=30 years |  | -0.71 | -0.50 | -0.56 |
|  |  | [-1.63, 0.19] | [-1.65, 0.64] | [-1.73, 0.60] |
| MX Birth and US 30+ years |  | 0.20 | 0.20 | 0.23 |
|  |  | [-0.76, 1.19] | [-0.99, 1.43] | [-0.97, 1.48] |
| County (Yuma) |  | -0.73 * | -2.08 *** | -2.10 *** |
|  |  | [-1.46, -0.02] | [-3.20, -1.08] | [-3.23, -1.09] |
| County (Santa Cruz) |  | -0.30 | -0.96 + | -0.97 + |
|  |  | [-1.13, 0.52] | [-2.13, 0.15] | [-2.15, 0.14] |
| Social Support:Hope |  |  |  | 0.01 |
|  |  |  |  | [-0.01, 0.02] |
| N | 213 | 213 | 213 | 213 |
| + p < 0.1, * p < 0.05, ** p < 0.01, *** p < 0.001 | | | | |
